# High-energy Trauma Precipitating Intramedullary Cavernous Malformation Hemorrhage – A Possible Underreported Mechanism

**DOI:** 10.7759/cureus.1092

**Published:** 2017-03-13

**Authors:** Pedro Aguilar-Salinas, Douglas Gonsales, Leonardo B Brasiliense, Eric Sauvageau, Ricardo A Hanel

**Affiliations:** 1 Lyerly Neurosurgery, Baptist Health, Jacksonville, Florida; 2 Division of Neurosurgery, University of Arizona, Tucson, AZ

**Keywords:** cavernous malformation, intramedullary, spinal cord, hemorrhage, trauma

## Abstract

Cavernous malformations are uncommon vascular lesions with an estimated prevalence of 0.5% in the general population. Intramedullary cavernous malformations (ICM) represent a rare subset of lesions, which account for approximately 5% of all cavernous malformations. The annual risk of hemorrhage in ICMs has been reported to range from 1.4 to 6.8%. Most patients are diagnosed with neurological dysfunction secondary to ICM hemorrhage and little is known about the inciting events that lead to hemorrhage. A few studies have suggested that minor and major trauma or even intense exertion may increase the risk of hemorrhage. We report the case of a 62-year-old male who developed progressive neurological deterioration following a motor vehicle accident. During work-up, an ICM was found at T4 and was surgically removed. At his 10-month follow-up, the patient had partially recovered, regaining motor strength in his right lower extremity, but had a persistent decrease in temperature and pinprick sensation on the left side starting at the T6 dermatome. We hypothesize that ICMs can rupture after high-energy impacts, such as the motor vehicle accident in our patient, and mechanical factors, such as trauma and stretching maneuvers, can play a role in the pathogenesis of ICM hemorrhage.

## Introduction

Cavernous malformations are benign vascular lesions with an estimated prevalence of 0.5% in the general population based on postmortem and imaging studies [1]. The histology of these lesions consists of sinusoidal blood cavities that lack the normal vessel layers, such as smooth muscle and adventitia, surrounded by a gliotic border with no nervous tissue involvement. The subset of intramedullary cavernous malformations (ICM) represents a rare entity, which occurs in approximately 5% of the cases with no gender predominance and can affect all age groups. The natural history of ICM remains largely unknown and published retrospective case series have reported an estimated annual hemorrhagic risk ranging from 1.4 to 6.8% with the most frequent location reported as the thoracic spinal segment in up to 77% of the cases [2]. Herein, we report an ICM found in a patient with progressive neurological deterioration after a high-energy motor vehicle accident, with this event as the possible triggering factor to hemorrhage.

## Case presentation

**Figure 1:**
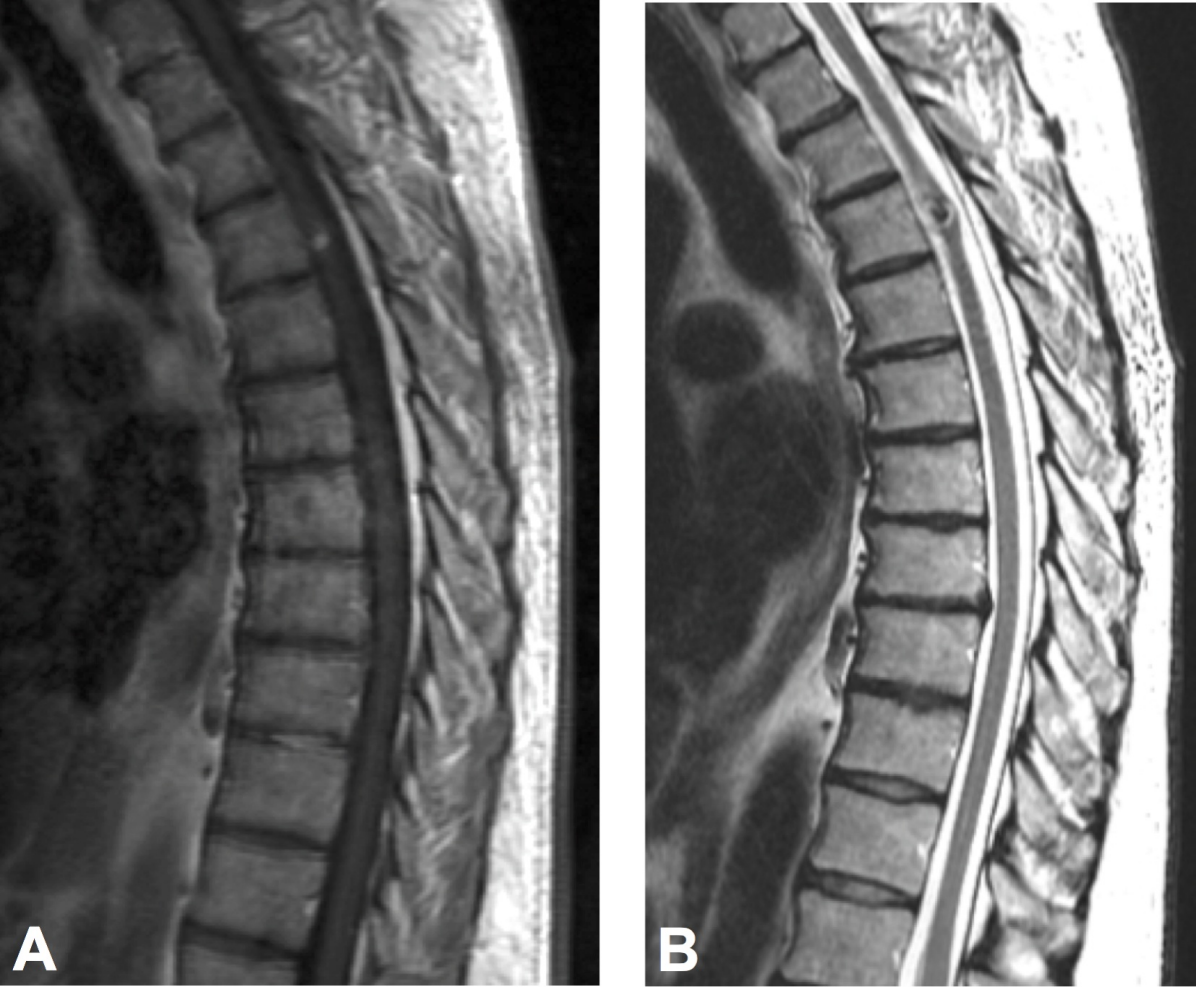
Thoracic spine MRI demonstrating an intramedullary cavernous malformation at the T4 level, Sagittal view in (A) T-1 sequence depicting a high-intense lesion and (B) T-2 sequence with hypointense signal and minimal edema.

A 62-year-old man presented to our service with a four-week history of progressive neurological deterioration after a high-energy impact in a car accident (curb weight car > 3,500 lbs at approximately 45 mph versus curb weight car < 2,600 lbs). Immediately after the event, the patient noticed a slight ecchymosis at the level of his left acromion, and over the next few weeks, he experienced progressive sensory changes on the left side of his body. Prior to the accident, the patient was completely asymptomatic. Examination revealed the loss of temperature and pinprick pain sensation from the T6 dermatome on his left side and right leg weakness. It is important to highlight that the patient was completely asymptomatic before the accident. MRI of the spine was performed and showed evidence of bleeding on the spinal cord at the T4 level, compatible with an intramedullary cavernous malformation (Figure 1).

**Figure 2:**
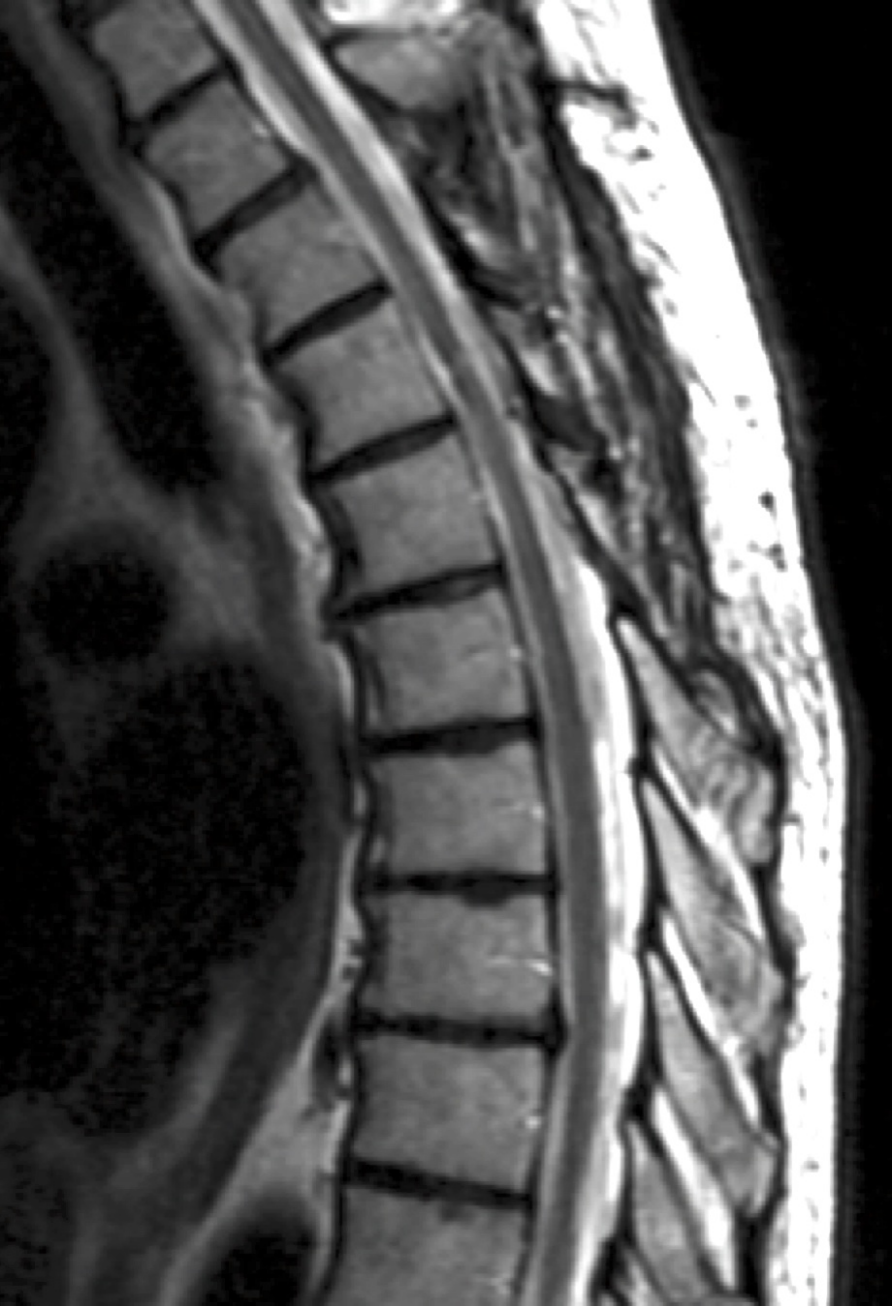
Thoracic spine MRI demonstrating no residual intramedullary cavernous malformation at the 10-month follow-up.

The patient underwent posterior T3-T5 laminoplasties and microsurgical excision of the ICM, which was confirmed by pathology. At his 10-month follow-up, the patient had recovered motor strength in his right lower extremity but had persistent left-sided decrease of temperature and pinprick sensation from T6 level. Thoracic spine MRI demonstrated no residual ICM (Figure 2).

## Discussion

Intramedullary cavernous malformations are low-flow lesions with dynamic episodes of thrombosis and recanalization, which result in subclinical events of microhemorrhage and chronic deposition of hemosiderin in the surrounding tissue. It has also been documented that these lesions may grow, regress, or arise de novo, but there is uncertainty about the phenomenon that causes the hemorrhage. Hypotheses have suggested that hemorrhage may be secondary to minor or major trauma, but the amount of energy required to disrupt the thin walls of the lesion remains unclear. It is also unknown whether the anatomical location plays a role in increasing the risk of bleeding. Labauge, et al. identified triggering factors for spinal cavernous hemorrhage in 26% (14/53) of their cases, which consisted of pregnancy, trauma, and strenuous activity [3]. In another case series, Liang, et al. found triggering factors of ICM hemorrhage in 17% (16/68) of their cases. Trauma was involved in seven cases and strenuous activity in nine [4]. McCormick, et al. presented a case series of six patients, in which two of them developed neurological dysfunction after a minor trauma in the upper back and strenuous exercise [5]. Anson, et al. reported in their case series an individual, a rodeo rider, with strenuous exercise activity that developed neurological dysfunction secondary to a cavernous malformation hemorrhage at the cervical level [6]. Tyndel, et al. reported the case of a pregnant woman with a calcified cavernous malformation at C5 that bled after moving furniture [7]. In another case report, Armstrong, et al. presented a patient with hematomyelia and acute neurological dysfunction after spinal chiropractic manipulation [8]. In our case, the patient presented with a four-week neurological deterioration after a high-impact car accident. Although the aforementioned cases series, as well as our case report, provide only a speculative mechanism of ICM hemorrhage, we believe the following factors could contribute to the rupture of the weak walls of the cavernous malformation in our patient: 1) the progression of symptoms began shortly after the car accident and followed a similar course to the progressive neurological deterioration in the largest ICM series, 2) the nature of the car accident in which a direct, rapid, and deceleration blunt trauma on his left acromion could have transmitted the energy to the spine as well as the simultaneous torsion when the car spun around (at least 120º), and 3) the low blood-flow of the ICM caused a slow bleed over weeks, which eventually led to a progressive neurological deterioration.

Recently, Badhiwala, et al. [2] published a meta-analysis of ICMs that included 632 patients from 40 studies. Interestingly, they found that less than 1% of the patients included in the study were asymptomatic. According to their review, the mean timeframe of preoperative symptoms was 29 months, which confirms that most cavernous malformations are found in acute or subacute clinical scenarios that can delay treatment. This also reflects the difficulty in the identification of triggering factors for rupture and progressive neurological dysfunction.

Regarding ICM treatment, it is highly accepted to intervene in all patients who develop symptoms. According to recent studies, the strongest predictor of a favorable neurological outcome is microsurgical resection within three months of symptom onset [9]. Additionally, this approach eliminates the risk of subsequent hemorrhage. On the other hand, conservative management of asymptomatic patients is still an issue of controversy due to the small sample of this subset reported in the literature and the lack of long-term clinical outcomes. However, we strongly suggest close monitoring for those patients as recently proposed by Zhang, et al. who reported an annual hemorrhagic risk of 3.9% of their cases in which conservative management was chosen [10].

## Conclusions

This report is limited by a single-case experience. However, it suggests that mechanical factors, such as trauma or stretching maneuvers, play an important role in the cavernous malformation rupture, and the clinician should be aware of these risks when an asymptomatic ICM is found. Blast injury and acceleration/deceleration have been described as the cause of microhemorrhage into the brain matter after different types of accidents. We propose closed trauma as a possible precipitating event for cavernous malformation hemorrhage.
